# Can USMLE and COMLEX-USA Scores Predict At-Risk Emergency Medicine Residents' Performance on In-Training Examinations?

**DOI:** 10.7759/cureus.58684

**Published:** 2024-04-21

**Authors:** Michael C Plewa, David J Ledrick, Kenneth Jenkins, Aaron Orqvist, Michael McCrea

**Affiliations:** 1 Emergency Medicine, Mercy Health - St. Vincent Medical Center, Toledo, USA; 2 Emergency Medicine, Ohio University Heritage College of Osteopathic Medicine, Athens, USA

**Keywords:** pearson‘s correlation, correlation, bland-altman, academic performance, in-training score, in-training, licensure examination, licensure, comlex level 2, united states medical licensing examination (usmle)

## Abstract

Purpose: The United States Medical Licensing Examination (USMLE) and Comprehensive Osteopathic Medical Licensing Examination (COMLEX) scores are standard methods used to determine residency candidates' medical knowledge. The authors were interested in using the USMLE and COMLEX part 2 scores in our emergency medicine (EM) residency program to identify at-risk residents who may have difficulty on the in-training exam (ITE) and to determine the cutoff values under which an intern could be given an individualized study plan to ensure medical knowledge competency.

Methods: The authors abstracted the USMLE and COMLEX part 2 scores and the American Board of Emergency Medicine (ABEM) ITE scores for a cohort of first-year EM residents graduating years 2010-2022, converting raw scores to percentiles, and compared part 2 and ABEM ITE scores with Pearson's correlation, a Bland-Altman analysis of bias and 95% limits of agreement, and ROC analysis to determine optimal the cut-off values for predicting ABEM ITE < 50^th^ percentile and the estimated test characteristics.

Results: Scores were available for 152 residents, including 93 USMLE and 88 COMLEX exams. The correlations between part 2 scores and ABEM ITE were r = 0.36 (95%CI: 0.17, 0.52; p < 0.001) for USMLE and r = 0.50 (95%CI: 0.33, 0.64; p < 0.001) for COMLEX. Bias and limits of agreement for both part 2 scores were -14 ± 63% for USMLE and 13 ± 50% for COMLEX in predicting the ABEM ITE scores. USMLE < 37^th^ percentile and COMLEX < 53^rd^ percentile identified 42% (N = 39) and 27% (N = 24) of EM residents, respectively, as at risk, with a sensitivity of 61% and 49% and specificity of 71% and 92%, respectively.

Conclusion: USMLE and COMLEX part 2 scores have a very limited role in identifying those at risk of low ITE performance, suggesting that other factors should be considered to identify interns in need of medical knowledge remediation.

## Introduction

Emergency medicine (EM) program directors and core faculty are interested in identifying residents at risk of poor academic performance early in their training, preferably early in the first post-graduate year (PGY1). The Council of Emergency Medicine Residency Directors (CORD) concluded that successful remediation is dependent on early identification of poor performance [1] and created both a remediation task force and a remediation consult service to address these issues [2]. A 2014 survey [3] found that 90% of EM programs had at least one resident undergoing remediation, with medical knowledge as the most common indication for remediation.

Performance on the American Board of Emergency Medicine (ABEM) in-training exam (ITE), which also predicts EM board certification success [4], is one method to assess academic progress and often the first indicator of identifying residents with knowledge deficits. Unfortunately, these results are not available until two months before the end of the PGY1 year. Aldeen et al. [5,6] demonstrated that EM faculty are only moderately able to predict resident performance on the ITE, with less accuracy for interns versus non-interns.

One of the primary factors in the residency selection process is a candidate’s performance on one of the licensure exams, either the United States Medical Licensing Examination (USMLE) or the Comprehensive Osteopathic Medical Licensing Examination - USA (COMLEX) [7]. Program directors often embrace the adage that the “best predictor of future performance is past performance” as licensure exam scores have been shown to correlate with ABEM ITE exam scores [8,9] and predict passing the ABEM qualifying exam following residency [10-13]. However, the relative importance of USMLE scores when EM program directors determine their applicant rank list has varied in published reports [14-16]. Multiple other specialties, including family medicine [17,18], general surgery [19], internal medicine [20,21], internal medicine/pediatrics [22], and obstetrics [23], have all examined the correlations between either licensure examinations or specialty ITE with specialty board certification examinations, with varying degrees of prediction [24].

Our primary goal was to determine the predictive value of the USMLE and COMLEX part 2 exam scores for the ABEM ITE scores for PGY1 residents in our EM program. Secondly, we sought to determine cutoff values from USMLE and COMLEX for predicting an ABEM ITE score below the 50th percentile to identify a PGY1 resident who would benefit from a medical knowledge remediation program before receiving the results of the ITE in the spring of the PGY1.

## Materials and methods

This was an IRB-approved (Mercy Health North Institutional Review Board 2020-5) retrospective analysis of all EM residents who graduated from 2010 to 2022 with complete data. Our program has 14 residents per year in a community urban teaching center. Although our residency historically participated only in the National Resident Matching Program, our hospital is also a core clinical training site for multiple osteopathic medical schools. As such, we have always interviewed and matched both allopathic and osteopathic medical students. Given that many osteopathic residents take both the COMLEX and USMLE exams, our program considers both exams.

Deidentified data from residency files were abstracted by a research assistant and reviewed by two of the authors to ensure a complete dataset that was corrected. We converted all part 2 USMLE, part 2 COMLEX, and the ABEM ITE exam scores into a percentile ranking appropriate for the year the test was taken. All data were entered into an Excel spreadsheet for analysis by a biostatistician.

Data were analyzed for association and agreement using several different approaches. For association, a Pearson correlation coefficient between the COMLEX or USMLE scores and the ABEM ITE scores was calculated with MedCalc® Statistical software (version 19.7.2; MedCalc Software Ltd., Ostend, Belgium). For agreement, we calculated bias and limits of agreement according to the methods of Bland et al. [25]. Bias is defined as the mean of the difference between scores, and the limits of agreement were defined as bias ± two standard deviations [25]. Additionally, a receiver operating curve (ROC) analysis was used to identify the optimal cut points for USMLE step 2 and COMLEX level 2 in predicting an ITE score below the 50th percentile for the year of training. Using these cutoff points, we calculated the percentage of EM1 residents at risk, the test characteristics, and the associated positive and negative likelihood ratios (LR) and odds ratios.

## Results

Complete data were available for 152 of 165 EM residents, including 93 USMLE and 88 COMLEX exams. Seven (3.8%) were excluded for missing data, including two ABEM ITE, one USMLE, and four COMLEX exams. The correlations between part two scores and ABEM ITE were r = 0.36 (95%CI: 0.17, 0.52; p < 0.001) for USMLE part 2 and r = 0.50 (95%CI: 0.33, 0.64; p < 0.001) for COMLEX part 2.

The mean of the differences (bias) between USMLE or COMLEX and ABEM ITE was -14.4 percentile points and 13.0 percentile points, respectively. The limits of agreement were ± 63 percentile points for USMLE part 2 and ± 50 percentile points for COMLEX part 2, in predicting the ABEM ITE.

**Figure 1:**
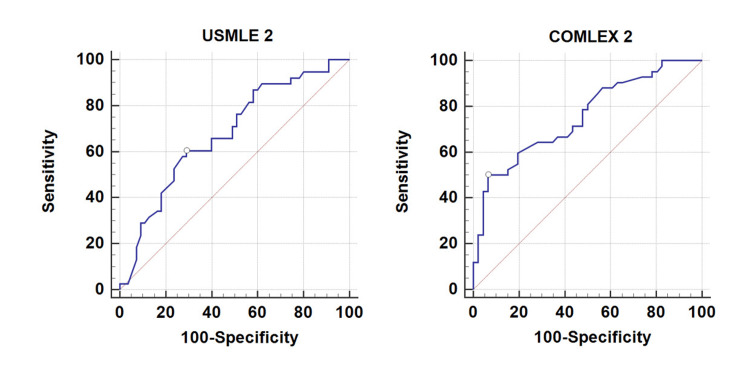
ROC curves for USMLE and COMLEX part 2 predicting ABEM ITE scores < 50% ABEM ITE: American Board of Emergency Medicine In-Training Exam; COMLEX-USA: Comprehensive Osteopathic Medical Licensing Examination - USA; ROC: receiver operating curve; USMLE: United States Medical Licensing Examination

ROC analysis identified cut-points of USMLE < 37th percentile and COMLEX < 53rd percentile to optimize the prediction of ABEM ITE < 50th percentile (Figure 1). These cut-points identified 42% (N = 39) and 27% (N = 24) of EM residents, respectively, as being at risk, with positive predictive values of 95.8% and 66.7% and negative predictive values of 59.4% and 70.3%, respectively (Table 1).

**Table 1:** Predictive ability of USMLE and COMLEX scores to identify PGY1 residents at risk of scoring low on the ABEM ITE † A cut-point score was determined by the receiver operating characteristic methodology to maximize the sum of sensitivity and specificity to predict ABEM ITE < 50th percentile. The data have been represented as N, signifying the number per group, and percentiles. Values of p < 0.05 are considered significant. ABEM ITE: American Board of Emergency Medicine In-Training Exam; COMLEX-USA: Comprehensive Osteopathic Medical Licensing Examination - USA; PGY1: First post-graduate year; USMLE: United States Medical Licensing Examination

Level 2 Exam	USMLE (N = 93)	COMLEX (N = 88)
Correlation with ABEM ITE	r = 0.36 (95%CI: 0.17, 0.52; p < 0.001)	r = 0.50 (95%CI: 0.33, 0.64; p < 0.001)
Mean of differences (bias)	- 14.4 percentile points	13.0 percentile points
Limits of agreement	± 63 percentile points	± 50 percentile points
Cut-point to predict residents at risk of ABEM ITE < 50^th^ percentile†	< 37^th^ percentile	< 53^rd^ percentile
Area under ROC curve	0.68 (95% CI: 0.57, 0.77)	0.73 (95% CI: 0.63, 0.82)
No. residents identified as at-risk	39 (42%)	24 (27%)
Sensitivity	60.5%	48.8%
Specificity	70.9%	91.5%
Positive predictive value	95.8%	66.7%
Negative predictive value	59.4%	70.3%
Odds ratio for low ABEM using cut-point score	3.74 (95% CI: 1.56, 8.95)	14.33 (95% CI: 3.84, 53.52)

## Discussion

Our study was unique in several aspects. First, we used percentile scores instead of raw scores for each of the USMLE, COMLEX, and ABEM ITE exams, allowing comparison between exams and different examination years. Second, we assessed the relationship of the COMLEX with the ABEM ITE in 57.8% of our cohort, in addition to USMLE, which has been previously reported [9,10]. Last, we assessed associations with correlation coefficients, and agreement with the Bland-Altman analysis [25], between part two scores and ABEM ITE. Our findings confirm an association between part two exams and ABEM ITE scores but indicate that these correlations were moderate (r = 0.36 for USMLE and r = 0.50 for COMLEX), where correlation values > 0.50 are considered strong, 0.30-0.49 are considered moderate, and < 0.30 is considered low [26]. Correlations were lower in our cohort than those described by Hiller et al. (r = 0.58) [10] and Thundiyil et al. (r = 0.76) [13]. The Bland-Altman analysis identified bias, with USMLE on average 14.4 percentile points lower and COMLEX on average 13 percentile points higher than the ABEM ITE scores. The limits of agreement were wide for both USMLE and COMLEX exams for ABEM ITE. Within two SDs, the USMLE part 2 would underestimate or overestimate ABEM ITE by as much as 77.3 and 48.5 percentile points, respectively. COMLEX part 2 would underestimate or overestimate ABEM ITE by as much as 37 and 63 percentile points, respectively.

Significant ORs for both USMLE part 2 and COMLEX part 2 again validate the association with the ABEM ITE scores but do not assess their predictive nature. Instead, the LR were not very useful, with only a slight to moderate effect on the probability of poor ABEM ITE performance. The LR positive for both USMLE and COMLEX were low (2.08 and 5.73, respectively), whereas the LR negative for both exams was large (0.56, for both).

Using our USMLE and COMLEX cut-offs would have resulted in large percentages (27-42%) of our PGY1 class undergoing medical knowledge remediation. By comparison, the 2014 survey of 160 allopathic EM programs found only 4.4% of EM residents in remediation [3]. Having approximately one-third of PGY1 residents in an individualized remediation program might be considered a significant burden since a tailored curriculum specific to the learner might include assigning a faculty mentor, assigned readings and exams, and an altered schedule allowing for more study early in the program.

Other methods should be considered in identifying interns with medical knowledge deficits. Although Aldeen et al. found an overall 60% accuracy, they admit that only 23% of faculty could correctly identify those residents scoring significantly below their class mean [5]. Goyal et al. discussed a variety of medical knowledge assessment tools, including USMLE, ABEM ITE, question bank scores, performance on virtual or simulation scenarios, oral exams, direct observation of skills, and objective structured clinical exams, among others [27].

This study had several limitations. Our sample was from a single program, and the results may not apply to programs with different selection or advancement criteria. Our sample size was modest but similar in size to that of Thundiyil et al. [13] and larger than that of Hiller et al. [10]. We also did not have complete data on 13 of 265 residents, though there was no clear pattern to the missing numbers. We did not include USMLE step 1 or COMLEX level 1 exam scores since these are now pass/fail as of 2022 and will do little to aid programs in assessing performance. We did not include part three exams since these are usually taken during the PGY1 and therefore irrelevant to our purpose.

## Conclusions

The USMLE and COMLEX exams are among the most standardized and universally applied measurements of medical knowledge competency. Our results concur with prior studies in that the USMLE and COMLEX part 2 exam scores do correlate with performance on ABEM ITE scores. However, we find this association is imperfect and demonstrates substantial bias and inadequate agreement. Because these scores have poor predictive value, and likely overestimate the number of interns in need of early academic intervention, programs should consider using alternative evaluation methods to be more specific in identifying those requiring additional resources.
